# Influenza A Virus Hemagglutinin is Required for the Assembly of Viral Components Including Bundled vRNPs at the Lipid Raft

**DOI:** 10.3390/v8090249

**Published:** 2016-09-10

**Authors:** Naoki Takizawa, Fumitaka Momose, Yuko Morikawa, Akio Nomoto

**Affiliations:** 1 Laboratory of Virology, Institute of Microbial Chemistry (BIKAKEN), 3-14-23 Kamiosaki, Shinagawa-ku, Tokyo 141-0021, Japan; anomoto@bikaken.or.jp; 2 Kitasato Institute for Life Sciences and Graduate School for Infection Control, Kitasato University, 5-9-1 Shirokane, Minato-ku, Tokyo 108-8641, Japan; fmomose@lisci.kitasato-u.ac.jp (F.M.); morikawa@lisci.kitasato-u.ac.jp (Y.M.)

**Keywords:** influenza virus, lipid raft, virus budding, viral genome packaging, hemagglutinin

## Abstract

The influenza glycoproteins, hemagglutinin (HA) and neuraminidase (NA), which are associated with the lipid raft, have the potential to initiate virion budding. However, the role of these viral proteins in infectious virion assembly is still unclear. In addition, it is not known how the viral ribonucleoprotein complex (vRNP) is tethered to the budding site. Here, we show that HA is necessary for the efficient progeny virion production and vRNP packaging in the virion. We also found that the level of HA does not affect the bundling of the eight vRNP segments, despite reduced virion production. Detergent solubilization and a subsequent membrane flotation analysis indicated that the accumulation of nucleoprotein, viral polymerases, NA, and matrix protein 1 (M1) in the lipid raft fraction was delayed without HA. Based on our results, we inferred that HA plays a role in the accumulation of viral components, including bundled vRNPs, at the lipid raft.

## 1. Introduction

The influenza A virus genome consists of eight single-stranded negative-sense viral RNA (vRNA) segments. This RNA genome interacts with a heterotrimeric viral RNA polymerase (PB2, PB1, and PA) complex and nucleoprotein (NP) to form the viral ribonucleoprotein complex (vRNP). The viral envelope surrounding vRNPs consists of a lipid bilayer containing three viral transmembrane proteins, hemagglutinin (HA), neuraminidase (NA), and matrix protein 2 (M2). HA functions in receptor binding and membrane fusion. NA cleaves the terminal sialic acids from the host cell to facilitate progeny virus release. M2 acts as a proton-selective ion channel and mediates membrane scission. Inside the viral envelope, matrix protein 1 (M1) is attached to the lipid envelope to stabilize virion structures.

The replication of vRNA and messenger RNA (mRNA) transcription occurs in the nucleus. Translated viral polymerases and NPs are imported into the nucleus to form vRNPs, and newly synthesized vRNPs are exported from the nucleus to the cytoplasm via a CRM1-dependent pathway [1,2,3]. The vRNPs are transported to the apical plasma membrane through interactions with recycling endosomes via Rab11 [4,5,6]. The eight individual segments are bundled after nuclear export [7,8,9]. To be an infectious virion, all eight segments are needed for incorporation in a virion. Recent studies favor the selective packaging model in which influenza virions selectively incorporate a set of the eight distinct vRNAs [10,11]. Each vRNA segment contains a unique “packaging signal” located in the terminal noncoding to coding regions at both the 3′ and 5′ ends [12].

Progeny virion budding occurs at membrane lipid rafts [13,14,15,16]. The lipid rafts are dynamic assemblies of cholesterol, sphingolipids, and phospholipids containing saturated fatty acids. HA and NA are incorporated in the lipid raft fraction and accelerate their apical transport through clustering of lipid rafts [17]. The association between HA and the lipid raft is important for the virus life cycle because mutations in the HA transmembrane domain that reduce raft association impair viral replication [13,16]. Several studies using a plasmid-based virus-like particle (VLP) system have shown that HA, NA, M2, and membrane-tethered M1 can mediate VLP budding [14,18,19]. Additionally, Chlanda et al. showed that HA, NA, M1, and M2 are all necessary for the assembly and release of VLPs similar to that of virions [20]. M1 is associated with lipid raft domains in infected cells and is unable to initiate VLP budding without membrane targeting [16,18]. The expression of HA and/or NA recruits M1 to lipid raft membrane fractions [21]. M2 is a cholesterol binding protein and colocalizes with lipid raft domains in infected cells [22]. It is speculated that M1 mediates the recruitment of M2 to lipid raft domains in infected cells because the M2 cytoplasmic tail can interact with M1 [22,23,24]. Based on these results, the following budding model has been proposed [25]; (1) membrane curvature occurs owing to HA and NA clustering in the lipid raft domain; (2) the budding virion is elongated by M1 polymerization; and finally, (3) membrane scission occurs by M2 at the budding neck.

The role of viral components in vRNA packaging is still unclear. It is speculated that progeny vRNPs are recruited to the budding site via vRNA binding to M1, because it is recruited to lipid raft domains by HA and/or NA, and the deletion of the cytoplasmic tail of HA and NA affects vRNP packaging to progeny virions [21,26]. However, the precise role of M1 during vRNP recruitment and packaging is currently unknown. In addition, it is not clear whether segmented genome bundling is necessary for progeny virion production. The cytoplasmic tail of M2 is also thought to be involved in the efficient packaging of the viral genome [27]. However, its precise role is also unknown. In this study, we focused on HA to analyze the budding of progeny virions and assembly of viral components, including vRNPs in the lipid raft. We show that HA is necessary for efficient progeny virion production and for the accumulation of viral components at the lipid raft. Our results provide mechanistic insight into the assembly of viral components and the budding of progeny virions containing all segments.

## 2. Materials and Methods

### 2.1. Vectors and Antibodies

Viral protein expression vectors (pcDNA762(PB2), pcDNA774(PB1), pcDNA787(PA), pCAGGS-WSN-NP0/14, and pEWSN-HA) and vRNA expression vectors (pPolI-WSN-PB2, pPolI-WSN-PB1, pPolI-WSN-PA, pPolI-WSN-HA, pPolI-WSN-NP, pPolI-WSN-NA, pPolI-WSN-M, and pPolI-WSN-NS) for the reverse genetics system were kindly provided by Dr. Y. Kawaoka [28]. To prevent the trypsin-independent cleavage of HA of influenza A/WSN/33, a mutation that changes the NA residue at position 130 from arginine to asparagine was introduced to pPolI-WSN-NA [29,30]. To construct pPolI-WSN-HAstop, pPolI-WSN-HA-529-531A, and pPolI-WSN-NA-R130N, inverted polymerase chain reaction (PCR) was carried out using pPolI-WSN-HA and pPolI-WSN-NA as templates with specific primer sets. In pPolI-WSN-HAstop, premature termination codons were introduced at lysine 4 (AAA to TAA) and leucine 5 (CTA to TAA) from the first methionine in HA. In pPolI-WSN-HA-529-531A, mutations that change the HA residue at position 529 from isoleucine to alanine (ATT to GCT) and at position 530 from leucine to alanine (CTG to GCA) were introduced.

Rabbit polyclonal antibodies against PB2, PB1, PA, and NP were kindly provided by Dr. K. Nagata [31,32]. Rabbit polyclonal antibody against M1 was kindly provided by Dr. N. Kobayashi [33]. Mouse monoclonal antibody RA5-22 against HA (BEI Resources, Bethesda, MD, USA) was provided. Mouse monoclonal antibodies C179 against HA (Takara Bio Inc., Otsu, Japan), NS1-23-1 against NS1 (Santa Cruz Biotechnology, Santa Cruz, CA, USA), and Sheep polyclonal antibody against NA (R&D systems, Minneapolis, MN, USA) were purchased. Mouse monoclonal antibody mAb61A5 against NP was described previously [34].

### 2.2. Cells and Viruses

MDCK cells were grown in minimal essential medium (MEM) (Wako Pure Chemical Industries, Osaka, Japan) containing 10% fetal bovine serum. MDCK-F11-WT cells expressing Flag-tagged Rab11 (FLAG-Rab11) [5] and HEK293T cells were grown in Dulbecco’s modified Eagle’s medium (DMEM) with high glucose (Sigma-Aldrich, St. Louis, MO, USA) containing 10% fetal bovine serum. To establish MDCK cells expressing HA (MDCK-HA), MDCK cells were transfected with pEWSN-HA and pCI-neo (Promega, Fitchburg, WI, USA). The transfected cells were selected under 500 µg/mL antibiotic G418 (Nacalai Tesque, Kyoto, Japan) and G418-resistant cell focuses were picked up after one week. HA expression was confirmed by western blotting and indirect immunofluorescence using the mouse anti-HA monoclonal antibodies RA5-22 and C179, respectively.

Mutant viruses were generated by a reverse genetics approach [28]. HEK293T cells were transfected with viral protein expression vectors (PB1, PB2, PA, NP, and HA) and viral RNA expression vectors. After 24 h post-transfection, the cell culture medium was changed to OPTI-MEM I (Life Technologies, Gaithersburg, MD, USA) containing 0.6 µg/mL TPCK-trypsin (Sigma-Aldrich). After incubation for 24 h, the cell culture supernatant was collected and used for virus amplification. HA mutant viruses were grown in MDCK-HA cells. The virus titer was determined by a plaque assay using MDCK-HA cells. A WSN virus that was mutated to prevent trypsin-independent cleavage of HA (WSN NA R130N virus) was used as the wild type (WT) virus.

### 2.3. Virus Concentration

MDCK cells were infected with each virus at a multiplicity of infection (MOI) of 1 for 1 h at 37 °C, and incubated in MEM at 37 °C for 23 h. The supernatant was collected, and cell debris was removed by low-speed centrifugation and filtration through a 0.45 µm filter (EMD Millipore, Billerica, MA, USA). The pre-cleared supernatant was layered onto a 30% sucrose cushion in 20 mM Tris-HCl (pH 7.9), 100 mM NaCl, and 1 mM EDTA buffer and centrifuged at 130,000 *g* using an SW55 rotor for 1.5 h. The pellet was suspended in phosphate-buffered saline (PBS) (-) or cell lysis buffer (20 mM Tris-HCl (pH 7.9), 100 mM NaCl, 1 mM EDTA, and 0.25% SDS). The vRNA was extracted with phenol/chloroform.

### 2.4. Western Blotting

The infected cells were suspended in cell lysis buffer and homogenized by passing through a syringe with a 23-gauge needle. The lysate was cleared by centrifugation at 15,000 *g* for 5 min at 4 °C. The supernatant was collected and used for western blotting. Viral proteins in the cell lysate and virions were detected by western blotting using the LAS4000 (GE Healthcare, Milwaukee, WI, USA). Band intensity was measured using ImageJ [35] and standard curves were obtained to semi-quantify the relative amount of viral proteins.

### 2.5. Primer Extension Assay

A primer extension assay was performed as described previously [33]. The total RNA from infected cells was extracted with ISOGEN reagent (NIPPON GENE, Tokyo, Japan). The [^32^P]-labeled oligonucleotides hybridized to each vRNA segment were mixed and used for the assay. The oligonucleotide sequences used for the primer extension assay to detect vRNAs were Seg1v (5′-ATTCAACTACAACAAGGCCA-3′), Seg2v (5′-GATGAGGATTACCAGGGG-3′), Seg3v (5′-GTACTGTGTTCTTGAGATAGG-3′), Seg4v (5′-GAGTTCTACCACAAGTGTGA-3′), Seg5v (5′-GGGAATACAGAGGGGAGAA-3′), Seg6v (5′-AGGGCTAGACTGTATAAGG-3′), Seg7v (5′-CGTCGCTTTAAATACGGTTTG-3′), and Seg8v (5′-TGAAGATAACAGAGAATAGT-3′) and the expected transcript lengths were 350, 468, 593, 300, 230, 200, 158, and 133 nucleotides, respectively. The oligonucleotide sequences used to detect positive-strand viral RNAs were Seg4m/c (5′-CCGTTGTGGCTGTCTTCGA-3′), and Seg7m/c (5′-TCCCCTTAGTCAGAGGTGAC-3′) and the expected transcript lengths were 199 and 198 nucleotides, respectively. [^32^P]-labeled products were visualized using the imaging analyzer Typhoon 9400 (GE Healthcare).

### 2.6. qPCR Assay

cDNA was synthesized from extracted RNA with uni-12 primer (5′-AGCRAAAGCAGG-3′) using ReverTra Ace (TOYOBO, Osaka, Japan). The synthesized cDNA was mixed with specific primer set and Thunderbird SYBR qPCR mix (TOYOBO). qPCR reaction was performed using Thermal Cycler Dice Real Time System TP800 (TaKaRa, Shiga, Japan). The oligonucletotide sequences used to detect vRNAs and control 18S rRNA were 5′-AGCAAGCCGTGGATATTTGC-3′ and 5′-TGAAGATTGCCCGTAAGCAC-3′ for segment 1, 5′-TGTTCAGCAAACACGAGTGG-3′ and 5′-TGTGTTGGCCAATGCTGTTG-3′ for segment 2, 5′-TTGAGGTCGCTTGCAAGTTG-3′ and 5′-TGTTCAATTGGAGCCGCATC-3′ for segment 3, 5′-GGCATCATCACCTCAAACGCGTC-3′ and 5′-ATCCTCAATTTGGTACTCCTGACA-3′ for segment 4, 5′-TAATGGTGACGATGCAACGG-3′ and 5′-ATTCCTGTGCGAACAAGAGC-3′ for segment 5, 5′-ACAATTGGCACGGTTCGAAC-3′ and 5′-AGCTGCCTGTTCCATCTTTG-3′ for segment 6, 5′-AGAGGGAGATAACATTCCATGGGGC-3′ and 5′-TGTTCACAGGTTGCGCATACCAGGC-3′ for segment 7, 5′-TTGGCATGTCCGCAAAAGAG-3′ and 5′-TTCGATGTCCAGACCAAGAGTG-3′ for segment 8, and 5′-CGGCGACGACCCATTCGAAC-3′ and 5′-GAATCGAACCCTGATTCCCCGTC-3′ for 18S rRNA.

### 2.7. Indirect Immunofluorescence

An indirect immunofluorescence assay was performed as described previously [33]. Mouse anti-HA monoclonal antibody C179, mouse anti-NP monoclonal antibody mAb61A5, rabbit anti-NP polyclonal antibody, sheep anti-NA polyclonal antibody, rabbit anti-M1 polyclonal antibody, and mouse anti-NS1 monoclonal antibody NS1-23-1 were used for viral protein immunostaining. Alexa Fluor 488 conjugated anti-mouse Ig, Alexa Fluor 594 conjugated anti-mouse Ig, Alexa Fluor 594 conjugated anti-rabbit Ig, and Alexa Fluor 488 conjugated anti-sheep Ig (Thermo Fisher Scientific, Waltham, MA, USA) were used for visualization. Specimens were observed using an LSM 5 confocal microscope (Carl Zeiss, Jena, Germany) or a DMI6000 B microscope (Leica Microsystems, Wetzlar, Germany). The Pearson correlation coefficient (PCC) was calculated using the Coloc2 plugin in Fiji/ImageJ.

### 2.8. Electron Microscopy

MDCK cells were fixed with 2.5% glutaraldehyde in 0.1 M cacodylate. The fixed cells were treated with 2% osmium tetroxide for 1 h at 4 °C, and were stained with uranyl acetate. Ultra-thin sections were examined with the electron microscope JEM-1200EX II (JEOL, Tokyo, Japan).

### 2.9. Lipid Raft Fractionation

Sample preparation and a floatation analysis were performed as described by Ohkura et al. [17], Ali et al. [21], and Carrasco et al. [36], with some modifications. MDCK cells (1 × 10^6^ cells in 60 mm dish) were infected with each virus at an MOI of 1 for 1 h at 37 °C. The infected cells were collected, and resuspended in 50 mM Tris-HCl (pH 7.9), 150 mM NaCl, 1 mM EDTA, and 1 mM DTT buffer. The cells were sonicated, and TritonX-100 (TX-100) was added (final concentration, 0.5%). The cells were incubated at 0 °C or 37 °C for 30 min, and unbroken cells and nuclei were removed by low-speed centrifugation at 500 *g* for 5 min at 4 °C. The resulting supernatant (90 µL) was dispersed into 720 µL of 80% (*w*/*v*) sucrose in PBS(-), transferred to an ultracentrifuge tube, sequentially overlaid with 2.5 mL of 60% and 1.1 mL of 10% sucrose in PBS(-), and centrifuged at 100,000 *g* for 16 h at 4 °C. After centrifugation, 0.5 mL fractions were collected from the top of the gradient. The detergent insoluble glycolipid complex associated proteins were collected in top fractions (fraction 2 and 3; layer of 10%-55% sucrose fractions).

## 3. Results

### 3.1. HA Is Necessary for Efficient Virion Production

**Figure 1:**
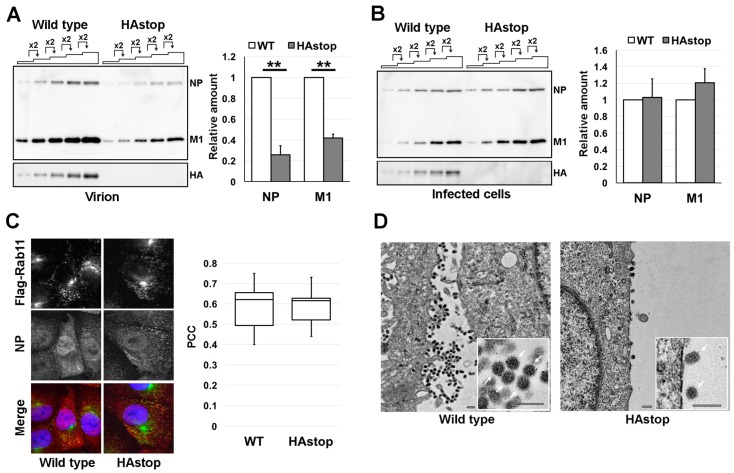
Decrease in virion production from cells infected with HAstop virus. (**A**) Detection of viral proteins in virions from cells infected with the HAstop virus. Wild-type (WT) and HAstop viruses were infected to MDCK cells at a multiplicity of infection (MOI) of 1. The virions were concentrated from the supernatant of infected cells at 24 hpi, and nucleoprotein (NP), matrix protein 1 (M1), and hemagglutinin (HA) were detected by western blotting. The ratio of the loaded sample volumes was 1:2:4:8:16. The band intensities of NP and M1 in the WT virus and HAstop virus were measured and the relative amount of each viral protein was semi-quantified from the standard curves. The graph indicates average values and standard deviations of three independent experiments. ** *p* < 0.01 by Student’s *t*-tests; (**B**) The detection of viral proteins in cells infected with the HAstop virus. The infected cell lysate was prepared at 10 hpi and NP,M1, and HA were detected by western blotting. The ratio of loaded sample volumes was 1:2:4:8:16. The band intensities of viral proteins were measured and the relative amount of each viral protein was semi-quantified from the standard curves. The graph indicates average values and standard deviations of three independent experiments; (**C**) The localization of viral ribonucleoprotein complex (vRNP) in cells infected with the HAstop virus. MDCK-F11-WT cells were infected with WT or HAstop virus at an MOI of 1. At 12 hpi, NP and FLAG-Rab11 were visualized. The Pearson correlation coefficient (PCC) between pixel intensity of NP staining and FLAG-Rab11 staining was calculated. The distribution of PCC from 10 independent cells in the same well infected with each WT and HAstop virus is indicated in the box plot; (**D**) Defective progeny virion production from cells infected with the HAstop virus. The MDCK cells infected with the WT virus or HAstop virus were fixed at 12 hpi and the cell surfaces were observed by thin-section electron microscopy. Scale bars: 500 nm.

To dissect the requirements for HA for progeny virion production, we generated recombinant viruses that did not express HA by introducing premature nonsense codons (HAstop virus). We made recombinant wild type and HAstop viruses that have a mutation in NA to prevent trypsin-independent cleavage of HA. This wild type virus is not capable of multi-cycle infection without trypsin (Supplemental Figure S1A). HAstop virus was infected to MDCK-HA cells and the titer of the supernatant at 48 hr post-infection (hpi) was 2.1 × 10^6^ PFU/mL. To analyze the production of progeny HAstop virions, the WT and HAstop viruses were used to infect MDCK cells at a multiplicity of infection (MOI) of 1 and the cell culture supernatants were collected at 24 hpi. About 70% of cells were infected (1 MOI) and the ratio of cells infected with WT and HAstop virus was comparable (Supplemental Figure S1B). The virions in the supernatants were concentrated by ultracentrifugation and analyzed by western blotting. The amounts of NP and M1 were 74.4% ± 8.73% and 58.0% ± 3.63% less in the HAstop virions than in the WT virions, respectively (Figure 1A). The expression levels of the NP and M1 proteins in cells infected with the HAstop virus were comparable to those in WT-infected cells (Figure 1B). These results suggest that HA is required for efficient progeny virion production.

To rule out the possibility that vRNP transport is affected by the absence of the HA protein, the localization of vRNP and FLAG-Rab11 in cells infected with the HAstop virus was observed by confocal microscopy and PCC between cells infected with WT and HAstop virus was calculated. We utilized MDCK cells that expressed FLAG-Rab11 (MDCK-F11A-WT) and the anti-NP monoclonal antibody preferentially bound to NP in RNP form [5,34]. The NP antigen, which most likely indicates progeny vRNP, was co-localized with FLAG-Rab11 in cells infected with the WT and HAstop viruses, indicating that the distribution of vRNPs in HAstop virus-infected cells was similar to that in WT virus-infected cells and the PCC was comparable (Figure 1C). To analyze whether virion budding is impaired by a lack of the HA protein, MDCK cells were infected with the WT or HAstop virus, fixed at 12 hpi, and examined by electron microscopy. Progeny virions of the HAstop virus were less frequently observed at the cell surface compared to those of the WT virus (Figure 1D). The shape of progeny HAstop virions was apparently normal, compared with that of the WT virions (Figure 1D). These results suggest that particle production from cells infected with the HAstop virus was impaired.

### 3.2. HA Is Required for Efficient vRNA Packaging into Progeny Virions

**Figure 2:**
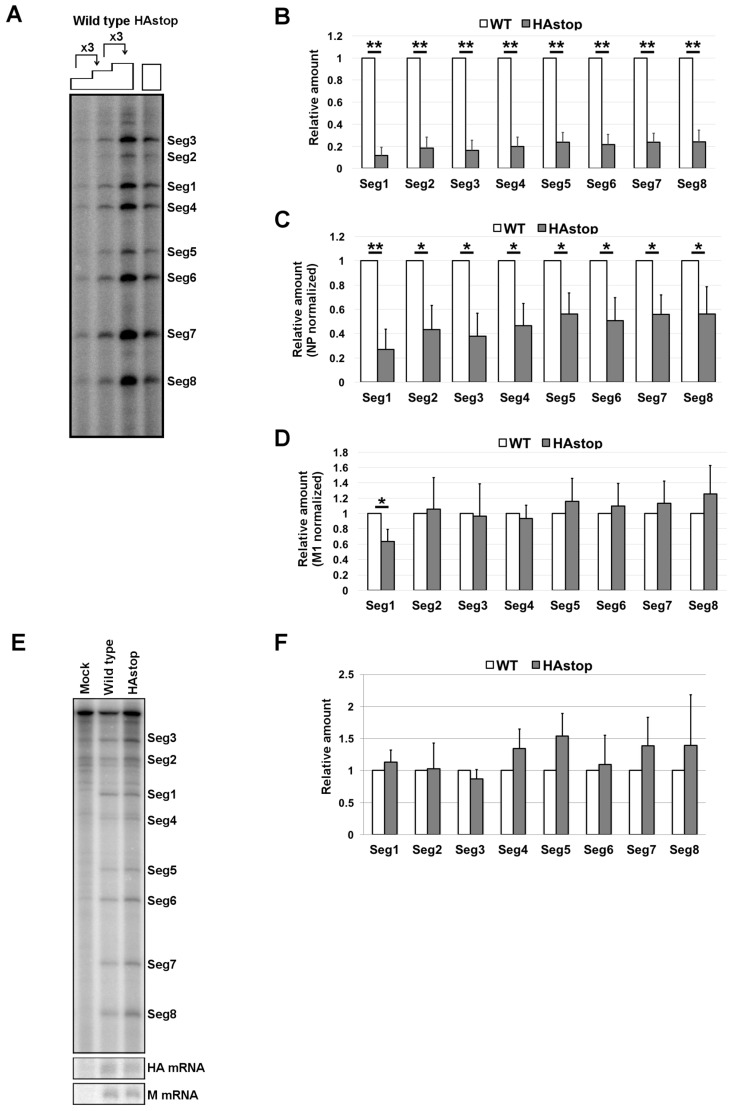
Decrease in packaged vRNPs in virions from cells infected with the HAstop virus. (**A**) Detection of each viral RNA (vRNA) segments in HAstop virions. The virions were concentrated from the supernatant of infected cells at 24 hpi. The vRNA was extracted from the virions and primer extension assay was performed; (**B**–**D**) Relative amount of vRNA in HAstop virions. The vRNA was extracted from the same samples used for western blotting in Figure 1A and the amount of each vRNA segment was determined by qPCR. The amount of each vRNA segment in the HAstop virus relative to that in the WT virus was calculated (**B**). The amounts of each vRNA segment were normalized by the amount of M1 (**C**) and NP (**D**) in the virions shown in Figure 1A. The graphs indicate average values with standard deviations of three independent experiments. ** *p* < 0.01 and * *p* < 0.05 by Student’s *t*-tests; (**E**) Detection of each vRNA segment in cells infected with HAstop virus. Total RNA was extracted from the infected cells at 10 hpi and primer extension assay was performed using [^32^P]-labeled primer specific to each vRNA segment or to HA and M mRNA; (**F**) Relative amount of vRNA in cells infected with HAstop virus. The vRNA was extracted from the same samples used for western blotting in Figure 1B and the amount of each vRNA segment was determined by qPCR. The amount of each vRNA segment in cells infected with HAstop virus relative to that in cells infected with WT virus was calculated. The graph indicates average values with standard deviations of three independent experiments.

To analyze vRNA packaging in the HAstop virus, vRNA was purified from the virion fractions and subjected to a primer extension assay. All eight vRNA segments were detected at once using ^32^P-labeled specific primer mixtures. Non-specific bands were detected in total RNA from mock-infected cells, but these bands were reduced or not detected using total RNA from infected cells because binding affinity of labeled primers to vRNAs is higher than that to cellular RNAs. All eight vRNA segments were detected in HAstop virus (Figure 2A). The amount of vRNA in the supernatant from cells infected with HAstop virus was determined by qPCR. The amount of vRNA in the supernatant was approximately 80% lower than that from cells infected with WT virus (Figure 2B). To examine the packaging efficiency of vRNP into a virion, the amount of vRNA in the virions was normalized by the amount of M1 protein in the virions (the same sample used in Figure 1A). It is speculated that the amount of M1 in a WT and HAstop virion is comparable because the shape of both virions are comparable (Figure 1D). The amount of packaged vRNA was approximately 50% lower in HAstop virions than in WT virions (Figure 2C). The observed reductions were similar for the eight vRNA segments. To confirm that the vRNA quantification accurately reflects vRNA packaging efficiency, the band intensity was normalized by the amount of NP (Figure 2D). If the quantification of viral proteins and vRNA in virions is appropriate, the calculated ratio of vRNA to NP should be 1. The ratio of vRNA to NP was near 1 for the most of the segments (Figure 2D). To confirm the amount of vRNPs and viral mRNA in cells infected with HAstop virus, total RNA was purified from infected cells and subjected to primer extension and qPCR assay. The amounts of M mRNA, HA mRNA, and vRNAs were comparable in WT and HAstop virus infected cells (Figure 2E,F). These results suggest that the packaging efficiency of vRNPs is decreased in cells infected with the HAstop virus.

### 3.3. The Bundling of vRNP Segments Is Not Impaired in Cells Infected with the HAstop Virus

The efficiency of vRNA packaging in the HAstop virus was lower than that of the WT virus, but the packaging efficiency of each vRNA segment in the HAstop virus was comparable (Figure 2). It is possible that both empty particles and all-eight-segments-packaged particles are produced from HAstop virus infected cells. To test this possibility, the packaging of each segment in a virion was estimated based on the expression of each viral protein in infected cells. The progeny virions from cells infected with the HAstop virus were not infectious because of a lack of the HA protein on the virion surface. Thus, we generated and utilized infectious HAstop virions containing low amounts of HA in MDCK-HA cells. To analyze whether low HA expression in MDCK-HA cells can rescue the defects of particle production and vRNP packaging observed in MDCK cells infected with the HAstop virus, the amounts of viral proteins in the HAstop virion fractions produced from MDCK-HA cells were semi-quantified. The amount of HA protein in the supernatant from HAstop virus-infected MDCK-HA cells was about 95% lower than that from WT virus-infected MDCK-HA cells, whereas the amount of HA in MDCK-HA cells infected with HAstop virus was about 75% lower than that infected with WT virus (Supplemental Figure S2A,B). The amounts of NP and M1 in the supernatant from HAstop virus-infected MDCK-HA cells were about 60% lower than those from WT virus-infected MDCK-HA cells, respectively (Supplemental Figure S2A). The amounts of NP and M1 in the MDCK-HA cells infected with HAstop virus were comparable to that infected with WT virus (Supplemental Figure S2B). These results suggest that low expression of HA cannot fully rescue the virion production defect caused by a lack of the HA protein, but that progeny virus from MDCK-HA cells infected with the HAstop virus (HA-low virus) can be utilized for next assay to prove the requirement of HA for production of all-eight-segments-packaged particles. The stock HA-low virus titer used to next assay was 7.6 × 10^6^ PFU/mL.

Next, we analyzed whether all vRNP segments were co-packaged in the HA-low virus. MDCK cells were infected with the HA-low virus at an MOI of 0.1 to prevent multi-infection of two or more viruses to one cell and were immunostained for two different viral proteins (out of HA, NA, NP, M1, and NS1) at 12 hpi. The single- and double-positive cells were counted and the ratios of the number of double-positive cells to the total number of single- and double-positive cells are shown in Table 1. If the bundling of vRNP segments was impaired in the HAstop virus produced from MDCK-HA cells, the number of double-positive cells would be reduced in the next round of infection compared to that of WT-infected cells. Our results indicated no differences in the number of double-positive (NP plus NA, NP plus M1, NP plus NS1, NA plus M1, NA plus NS1, and M1 plus NS1) cells when they were infected with the WT virus and the HAstop virus produced from MDCK-HA (HA-low virus) (Table 1). This result suggests that the incorporation rate of the two vRNP segments tested in this experiment is comparable between the WT and HA-low virus, suggesting that the low level of HA protein was unlikely to have skewed the ratios of vRNP packaging. Taken together, these results suggest that the eight different vRNP segments are co-packaged in virions from HAstop-infected MDCK-HA cells, despite the decreased progeny virion budding and vRNP packaging.

### 3.4. The Lipid Raft Association of NP Is Reduced in Cells Infected with the HAstop Virus

**Figure 3:**
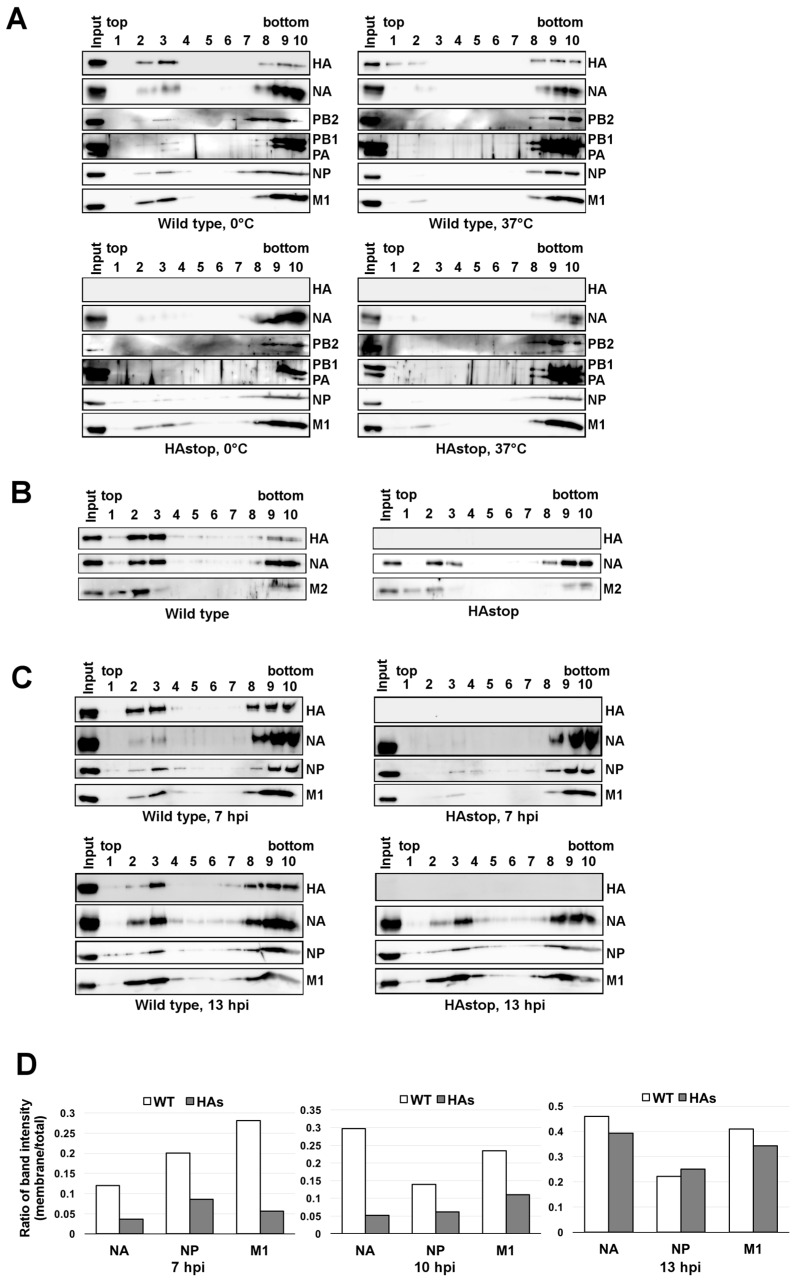
Decreased lipid raft associated NP in cells infected with the HAstop virus and non-raft HA virus. (**A**) Amount of viral proteins associated with the lipid raft in cells infected with the HAstop virus. The MDCK cells were infected with WT and HAstop virus, and were collected at 10 hpi. The cells were sonicated, treated with 0.5% Triton X-100 at 0 °C or 37 °C, and subjected to membrane flotation assays. HA, NA, PB2, PB1, PA, NP, and M1 in the fractions were detected by western blotting; (**B**) Amount of viral membrane proteins associated with the lipid raft. The MDCK cells were infected with WT and HAstop virus, and were collected at 10 hpi. The cell lysate was prepared, and subjected to membrane flotation assays. HA, NA, and M2 in the fractions were detected by western blotting; (**C**) Amount of viral proteins associated with lipid raft at early and late phase of infection. The MDCK cells were infected with WT and HAstop virus, and were collected at 7 and 13 hpi. The cell lysate was prepared, and subjected to membrane flotation assays. HA, NA, NP, and M1 in the fractions were detected by western blotting; (**D**) Relative amount of viral proteins in lipid raft fraction at 7, 10, and 13 hpi. The MDCK cells were infected with WT and HAstop virus, and the membrane flotation assays were performed using infected cell lysate prepared at 7, 10, and 13 hpi. NA, NP, and M1 in each fraction were detected by western blotting and the band intensities of viral proteins in each fraction were measured. The graph indicates average values of the ratio of the membrane-fraction band intensities (fractions 2 and 3) to the total band intensity (fractions from 1 to 10) of two independent experiments.

Since progeny virion budding occurs at membrane lipid rafts, it is possible that NA, M1, and progeny vRNPs did not sufficiently accumulate on the lipid raft in the absence of the HA protein. To test this hypothesis, we analyzed the association of viral polymerases, HA, NA, NP, and M1 with the lipid raft at 10 hpi by membrane flotation assays. Lipid rafts are insoluble in nonionic detergents such as TX-100, at 0 °C, but are soluble at 37 °C. Fractions of HA, NA, viral polymerases, NP, and M1 were observed in TX-100-insoluble membrane fractions in WT virus-infected cells at 0 °C (Figure 3A, fractions 2 and 3) but they became soluble at 37 °C (Figure 3A). These results confirmed the association of HA, NA, NP, and M1 with lipid rafts in WT virus-infected cells. Viral polymerases and NP were also recovered in the detergent-insoluble membrane fractions, indicating that vRNPs were associated with the lipid raft (Figure 3A). In contrast, for cells infected with the HAstop virus, a trace amount of vRNP components (viral polymerases and NP) was recovered in the detergent-insoluble membrane fractions. The amount of M1 in the fractions was comparable to that in cells infected with the WT virus. To analyze the association of viral membrane proteins, HA, NA, and M2, with the lipid raft in HAstop virus-infected cells, the membrane flotation assays were performed using WT and HAstop virus-infected cell lysate (Figure 3B). NA and M2 were still recovered in the detergent-insoluble membrane fractions, but the amount of NA and M2 in the fractions was clearly reduced compared to that in cells infected with the WT virus (Figure 3B). These results suggest that the accumulation of some viral components in the lipid raft was impaired in cells infected with the HAstop virus. Next, we analyzed the association of viral proteins with lipid raft at early (7 hpi) and late phase (13 hpi) of infection. The distributions of HA, NA, NP, and M1 in the fractions were analyzed by western blotting. The membrane-fraction band intensities and the total band intensity of NA, M1, and NP were measured and the ratio of membrane-fraction intensity to total intensity was calculated. At 7 and 10 hpi, the level of NA, NP, and M1 in the detergent-insoluble membrane fractions was about 60%–80% lower than that of WT fractions. In contrast, the level of NA, NP, and M1 in the fractions was comparable to that of WT fractions at 13 hpi (Figure 3C,D). These results suggest that the accumulation of viral components in the lipid raft was delayed in cells infected with the HAstop virus and the total number of viral components in lipid raft became comparable between cells infected with WT and HAstop virus at 13 hpi.

**Figure 4:**
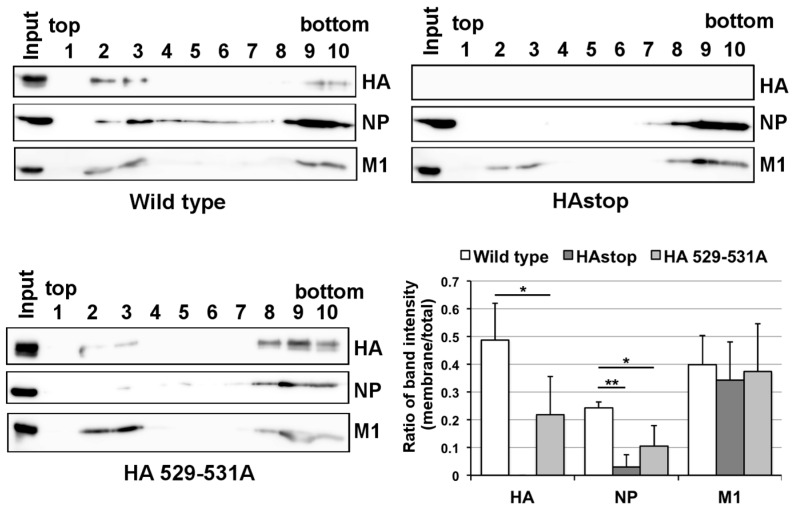
Amount of viral proteins associated with the lipid raft in cells infected with the non-raft HA virus. The MDCK cells were infected with the WT, HAstop, and HA 529–531A virus, and were collected at 10 hpi. The cell lysate was prepared, and subjected to membrane flotation assays. HA, NP, and M1 in each fraction were detected by western blotting and the band intensities of viral proteins in each fraction were measured. The graph indicates average values of the ratio of the membrane-fraction band intensities (fractions 2 and 3) to the total band intensity (fractions from 1 to 10) with the standard deviation of three independent experiments. * *p* < 0.05, ** *p* < 0.01 by Student’s *t*-tests.

To analyze whether the lipid raft association of HA is required for the association of vRNP with the lipid raft, we generated a recombinant virus that expresses non-raft-associated HA (HA 529–531A virus, residues 529–531 in H1 subtype numbering, corresponding to residues 530–532 in H3 subtype numbering) [16]. We performed membrane flotation assays using HA 529–531A virus-infected cell extracts. The distributions of HA, NP (as a vRNP representative), and M1 in the fractions were analyzed by western blotting. The level of HA 529–531A in the detergent-insoluble membrane fractions was much lower than that of WT fractions (Figure 4). Similarly, the level of NP in the lipid raft fractions in cells infected with HA 529–531A virus was very low, similar to that of the HAstop virus. In contrast, the amount of M1 in the fractions was comparable for cells infected with WT, HAstop, and HA 529–531A viruses. These results suggest that the lipid raft association of HA is necessary for vRNP recruitment to the lipid raft.

**Figure 5:**
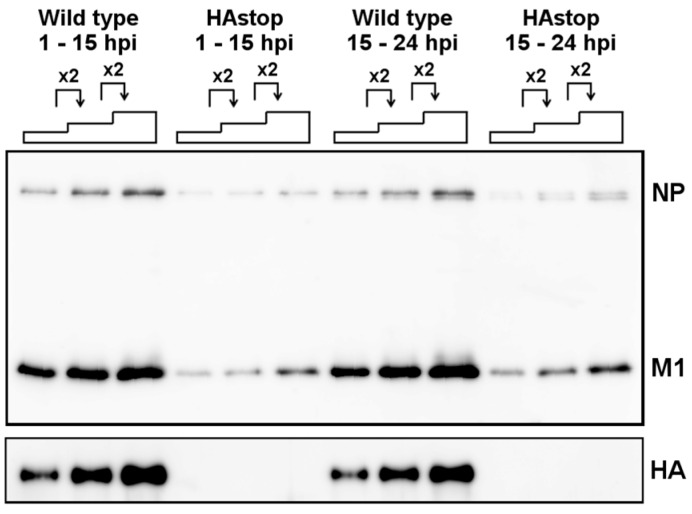
Decrease in virion production from cells infected with HAstop virus at both early and late phase of infection. MDCK cells were infected with WT and HAstop viruses. The supernatant of infected cells was collected at 15 hpi, and cells were suspended in MEM. The supernatant was also collected at 24 hpi. The virions were concentrated from the supernatant, and NP and M1 were detected by western blotting.

Our results show that the virion production is decreased and the association of viral components with lipid rafts is delayed in cells infected with HAstop virus. To analyze the relation between the decrease in virion production and the amount of viral proteins associated with lipid rafts in cells infected with HAstop, the number of virions in the supernatant from cells infected with WT and HAstop virus between 15 and 24 hpi was determined. The amounts of NP and M1 in the supernatant from cells infected with HAstop virus both between 1 and 15 hpi and between 15 and 24 hpi were decreased compared to that from cells infected with WT virus (Figure 5). This result suggests that the virion production is decreased from cells infected with HAstop virus even when the number of viral components associated with lipid rafts is comparable to that in cells infected with WT virus.

## 4. Discussion

NA is capable of binding to the lipid raft and co-expression of HA and NA induces their accumulation in lipid rafts and accelerates their apical targeting [17,37]. Our results suggest that the accumulation of NA in the lipid raft fraction was delayed in cells infected with the HAstop virus (Figure 3C,D). Previous report suggests that HA and NA mutually induce lipid raft clustering [17]. Thus, the accumulation of NA in the lipid rafts could be delayed without HA. The accumulation of NP in the lipid raft fraction was also delayed in cell infected with HAstop virus (Figure 3C,D). At 13 hpi, the levels of NA and NP associated with the lipid raft in cells infected with HAstop virus were comparable to those in cells infected with WT virus (Figure 3D). These results indicate that vRNPs could bind to the lipid raft without HA and the lipid raft accumulation of NA could rescue the binding of vRNPs to the lipid raft in HAstop virus infected cells. The accumulation of M1 in the lipid raft fraction was also delayed in cells infected with HAstop virus (Figure 3C,D). M1 is speculated to recruit vRNPs to budding site by binding to both NP and cytoplasmic tails of HA and NA [21,26]. Our results support the model that viral glycoproteins recruit M1 to the lipid rafts.

The virion production from cells infected with HAstop virus between 15 and 24 hpi was reduced compared to that with WT virus (Figure 5), whereas the levels of NA, NP, and M1 in the lipid raft were comparable at 13 hpi (Figure 3C,D). In addition, the initiation of budding from cells infected with HAstop virus looks still reduced at 12 hpi (Figure 1D). These results indicate that HA is necessary for both assembling viral components in lipid rafts and efficient budding of progeny virions. In cells infected with HAstop virus, the accumulation of NA in the lipid rafts could rescue the assembling other viral components in lipid rafts but could not rescue the budding defect. It is not known whether HA has an independent role for progeny virion budding or overexpression of NA can rescue the budding defect caused by lack of HA. HA and NA formed separate clusters and NA clusters are preferentially found on the spherically curved area whereas HA is preferentially found cylindrically curved sides of the filamentous virus like particle [20,38]. These reports indicate that HA and NA may have a different role of making membrane curvature of the virion. Thus, HA would have an independent role for initiation of budding process rather than the insufficient number of viral glycoproteins.

The transmembrane domain of HA and three palmitoylated cysteine residues (one located in the transmembrane domain and two in the cytoplasmic tail) are essential for the association between HA and the lipid raft [13,16]. The lipid raft association of HA is necessary for efficient virion production because mutations in the domain and residues failed to result in an association between HA and the lipid raft and attenuated viral replication [13,16]. In this study, we showed that the lipid raft association of HA is necessary for vRNP tethering to the lipid raft fraction (Figure 3). A previous study has shown that the lack of the cytoplasmic tail in HA is not crucial for virion assembly and that infectivity of the progeny virus is comparable to that of the WT virus [39]. This in turn suggests that not just the cytoplasmic tail but also other regions in HA are also involved in vRNP tethering to the lipid raft. Previous reports suggest that M1 and M2 are also necessary for the efficient packaging of vRNP and that all HA, NA, M1, and M2 are required for budding and release of VLP very similar to that of virions [20,26,27]. Viral glycoproteins may coordinate with M1, and M2 and facilitate the binding of vRNP to the lipid raft, rather than directly tethering the vRNP through the cytoplasmic tail.

It has been shown that vRNP segments are bundled after nuclear export [7,8,9] and are transported to the apical plasma membrane through interactions with recycling endosomes via Rab11 [4,5,6]. In contrast, HA does not localize at Rab11-positive vesicles [5]. These reports show that the transport mechanism of vRNP to plasma membrane is different from that of HA. In this context, we show that the bundling of vRNP segments was not impaired, even when HA expression was low (Table 1). We also show that the association of vRNP component proteins with lipid rafts at 10 hpi and the production of progeny virion were reduced by the lack of HA (Figure 1A and Figure 3A). Our results suggest that bundled vRNPs are associated with lipid rafts.

The incorporation of vRNP in the virion is not absolutely required for virion budding because the expression of viral membrane proteins alone can release VLP [14,18,19,20]. However, previous studies suggest that vRNPs are required for efficient virion budding, indicating that a large fraction of virions contain eight vRNP segments [10,11,38] and that mutations into important regions of the genome packaging diminish the total number of released virions [40,41]. In this study, we showed that in cells infected with the HAstop virus, the budding of progeny virions is reduced (Figure 1) and the association between NP and the lipid raft is also reduced (Figure 3A). We also showed that vRNA packaging into virions is reduced in cells infected with the HAstop (Figure 1 and Figure 2) but that the bundling of vRNP segments is not likely impaired in MDCK-HA cells infected with the HAstop virus (Table 1). These results raise the possibility that the recruitment of bundled vRNPs to the lipid rafts may be a trigger for efficient virion budding. The mechanism by which vRNPs regulate the initiation of the budding process needs to be elucidated in future studies.

## Figures and Tables

**Table 1:** The ratio of co-stained cells infected with wild type or HA-low virus.

		Correlating Protein Expression (%)			
	Virus	HA	NP	NA	M1
**NP**	WT	74.6 ± 8.35			
	HA-low ^a^	n.t.			
**NA**	WT	89.5 ± 8.87	85.0 ± 4.50		
	HA-low	n.t.	84.8 ± 5.65		
**M1**	WT	83.8 ± 7.13	73.1 ± 9.83	86.1 ± 6.84	
	HA-low	n.t.	76.7 ± 7.21	87.4 ± 4.20	
**NS1**	WT	n.t.	78.6 ± 9.61	88.1 ± 6.46	91.8 ± 5.43
	HA-low	n.t.	80.3 ± 7.00	88.9 ± 3.46	93.2 ± 3.11

HA: hemagglutinin; M1: matrix protein 1; NA: neuraminidase; NP: nucleoprotein; NS1: nonstructural protein 1; n.t.: not tested; a HA-low virus was derived from MDCK-HA cells infected with HAstop virus. MDCK cells were infected with wild type or HA-low virus at MOI of 0.1. At 12 hpi, cells were fixed and co-stained for indicated viral proteins. The stained cells were counted in independent 75 views (total 1500–4000 cells). The values represent the ratio of co-stained cells per all stained cells.

## Supplementary Materials

The following supplementary figures are available online at www.mdpi.com/1999-4915/8/8/249/s1, Figure S1: Infectivity of WSN NA R130N virus and HAstop virus, Figure S2: Decreased virion production from cells expressing low amounts of HA.
