# Supplementary material for: Influenza A Virus Hemagglutinin is Required for the Assembly of Viral Components Including Bundled vRNPs at the Lipid Raft
Source: Viruses. 2016 Sep 10;8(9):249. doi: 10.3390/v8090249 (PMC5035963; doi:10.3390/v8090249)
Supplement: Supplementary file 1 [file viruses-08-00249-s001.pdf]

Supplementary Materials: Influenza A Virus Hemagglutinin is Required for the Assembly of Viral Components Including Bundled vRNPs at the Lipid Raft

Naoki Takizawa, Fumitaka Momose, Yuko Morikawa and Akio Nomoto

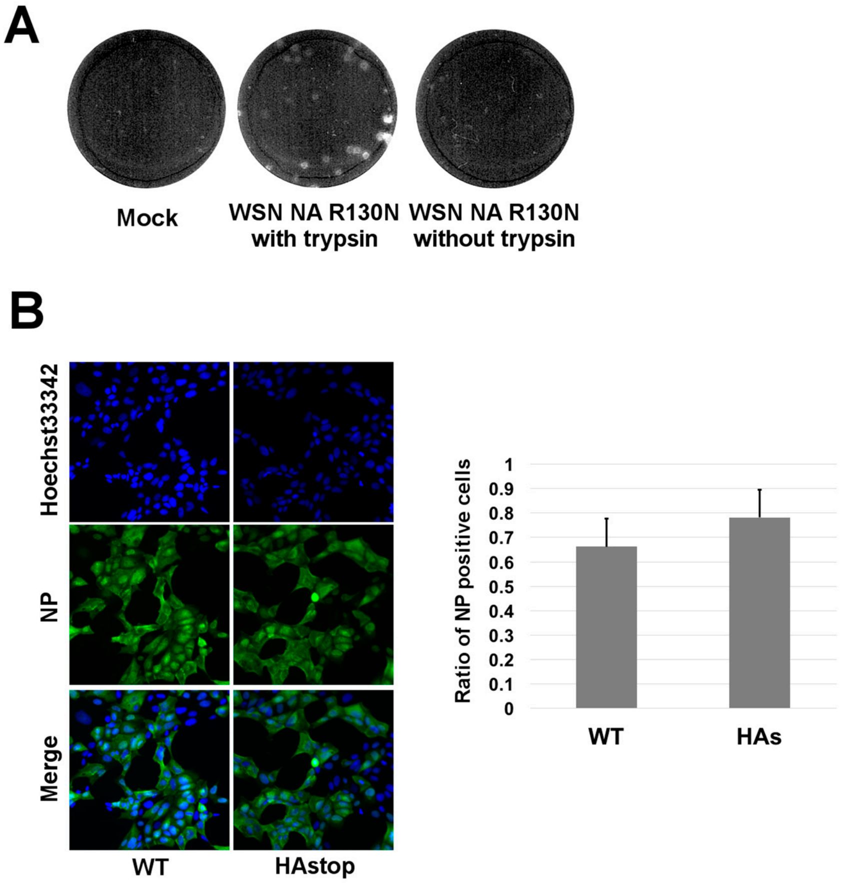

Figure S1. Infectivity of WSN NA R130N virus and HAstop virus (**A**) Trypsin dependent growth of WSN NA R130N virus. MDCK cells were infected or mock-infected with WSN NA R130N virus. After 1 hpi, MEM-0.8% agarose with or without 0.6 µg/ml TPCK-trypsin were overlaid to the infected cells. The cells were incubated at 37°C for 2 days, and stained with 1% amido black. (**B**) The ratio of infected cells at an MOI of 1 infection. MDCK cells were infected with wild type (WSN NA R130N) or HAstop virus at an MOI of 1. At 10 hpi, the cells were fixed and NP was visualized using specific antibody and Alexa Fluor 488 conjugated anti-mouse Ig. Nucleus was visualized using Hoechst33342. The graph indicates average values of the ratio of NP positive cells to total cells with standard deviation of 10 independent views (about 150 cells/ view).

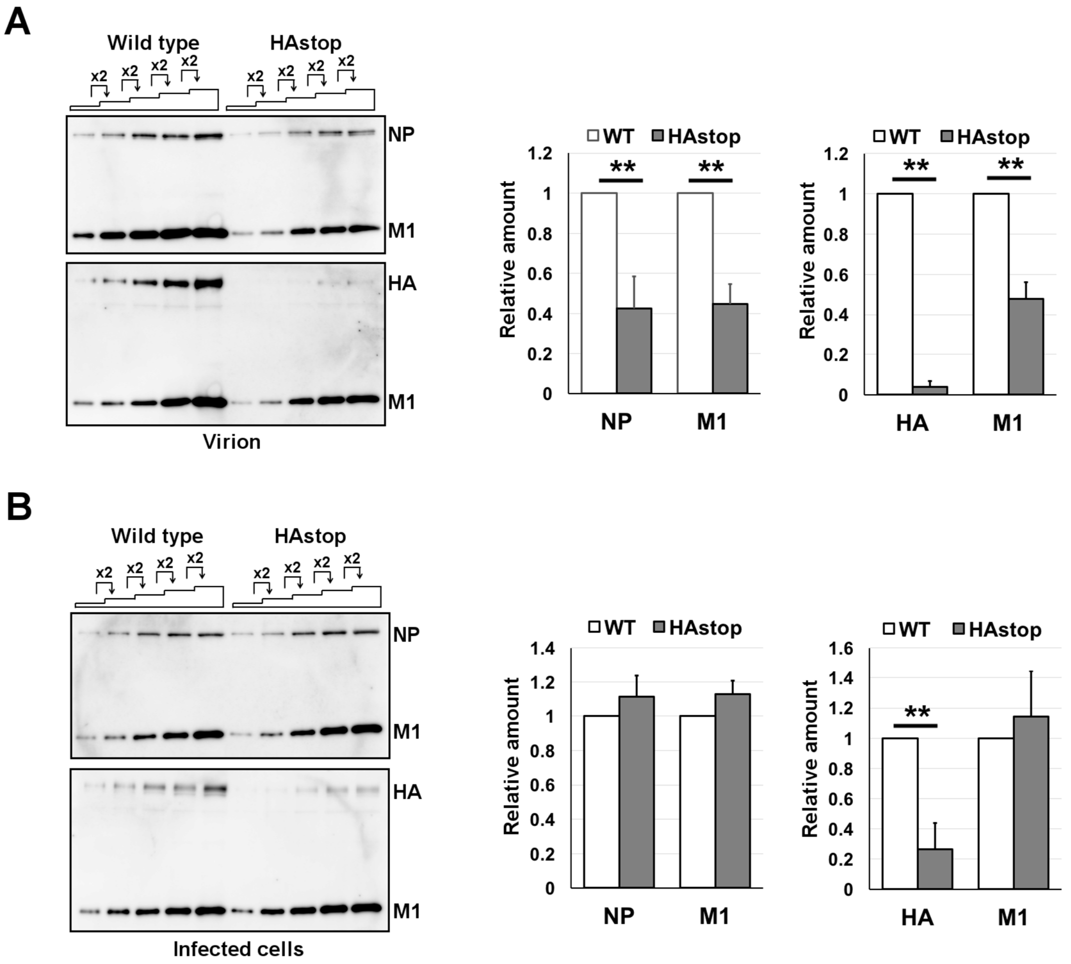

Figure S2. Decreased virion production from cells expressing low amounts of HA. The amount of viral proteins in virion from MDCK-HA cells infected with HAstop virus (**A**) and in HAstop virus infected MDCK-HA cells (**B**). WT and HAstop viruses were infected to MDCK cells at an MOI of 1. The virion was concentrated from the supernatant of infected cells at 24 hpi (**A**) or the infected cell lysate was prepared at 10 hpi (**B**). NP and M1 were detected by western blotting with anti-NP and anti-M1 antibody mixture followed by reprobing with anti-HA and anti-M1 antibody mixture. The ratio of the loaded sample volumes was 1:2:4:8:16. The band intensities of viral proteins were measured and standard curves were obtained. The amount of each viral protein in the HAstop virus relative to that in the WT virus was semi-quantified from the standard curves. The graph indicates average values and standard deviations of three independent experiments. **P < 0.01 by Student's t-tests.

© 2016 by the authors, licensee MDPI, Basel, Swizerland. This article is an open access article distributed under the terms and conditions of the Creative Commons Attribution (CC-BY) license (http://creativecommons.org/licenses/by/4.0/).
